# TAP-I Deficiency Presenting With Chronic Granulomatous Rubella Virus-Driven Cutaneous Ulceration: A Case Report and Scoping Literature Review

**DOI:** 10.1007/s10875-025-01919-6

**Published:** 2025-11-27

**Authors:** Mark J. Ponsford, Emily M. Carne, Kathryn Bramhall, Kristin Ladell, Ludmila Perelygina, Aung Saw, Kelly Miners, Sian Llewellyn-Lacey, Simon Kollnberger, Ian Tully, Sian Hughes, Hywel Williams, Manju Kalavala, Venetia Bigley, Daniel Farewell, David A. Price, Stephen L. Walker, Kathleen E. Sullivan, Stephen Jolles

**Affiliations:** 1 All Wales Syndrome Without A Name (SWAN) Clinic, University Hospital of Wales, Cardiff, UK; 2 Immunodeficiency Centre for Wales, University Hospital of Wales, Cardiff, UK; 3 Division of Infection and Immunity, School of Medicine, Cardiff University, Cardiff, UK; 4 Centers for Disease Control and Prevention, Division of Viral Diseases, Atlanta, GA USA; 5 Department of Medical Genetics, All Wales Medical Genomics Service, University Hospital of Wales, Cardiff, UK; 6 Department of Histopathology, University College London Hospitals NHS Foundation Trust, London, UK; 7 Division of Cancer and Genetics, School of Medicine, Cardiff University, Cardiff, UK; 8 Welsh Institute of Dermatology, University Hospital of Wales, Cardiff, UK; 9 Translational and Clinical Research Institute, School of Medicine, Newcastle University, Newcastle, UK; 10 Newcastle Hospitals NHS Foundation Trust, Newcastle upon Tyne, England UK; 11 Division of Population Medicine, School of Medicine, Cardiff University, Cardiff, UK; 12 Systems Immunity Research Institute, School of Medicine, Cardiff University, Cardiff, UK; 13 Hospital for Tropical Diseases, University College London Hospitals NHS Foundation Trust, London, UK; 14 Department of Dermatology, University College London Hospitals NHS Foundation Trust, London, UK; 15 Faculty of Infectious and Tropical Diseases, London School of Hygiene and Tropical Medicine, London, UK; 16 Division of Allergy and Immunology, The Children’s Hospital of Philadelphia, Philadelphia, PA USA

**Keywords:** TAP1, MHC 1, Immunodeficiency, Rubella

## Abstract

Autosomal recessive mutations in *TAP1, TAP2, TAPBP,* or *B2M*, are associated with major histocompatibility complex (MHC) class I deficiency. Individuals may present with granulomatous skin ulceration, but the underlying antigenic triggers remain largely unknown. We identified *TAP1* deficiency in a 32-year-old female referred with a 7-year history of localized skin ulceration. Histologic immunofluorescence revealed that rubella virus (RuV) infection was a likely driver of the associated inflammation, and modest clinical improvement was observed following topical calcineurin inhibition. To better define the natural history, clinical, and immunological manifestations of this condition, we also performed a scoping literature review. We identified 45 unique individuals from 36 reports with a combined follow-up duration of 1,184 patient years. Chronic necrotizing granulomatous skin lesions and childhood-onset bronchiectasis were common. Five deaths were reported (median age 36 years), typically linked to respiratory complications. Phenotypic heterogeneity was evident, with at least four individuals reaching adulthood without clinical symptoms. Diagnostic delay frequently exceeded a decade amongst symptomatic individuals, with misdiagnosis of granulomatous disease prompting systemic immunosuppression and infection-related morbidity. The presence of an abnormal CD8^+^ T-cell count or a history of consanguinity offered low sensitivity for MHC I deficiency (~ 50%), indicating a low threshold for further investigation is required for correct diagnosis. Graphical review of case reports identified morphologically similar lesions in other MHC I-deficient individuals. These findings suggest that the phenomenon of MHC I deficiency is underreported and that diagnosis should prompt testing for RuV.

**Supplementary Information:**

The online version contains supplementary material available at 10.1007/s10875-025-01919-6.

## Introduction

Individuals with inborn errors of immunity often present following diagnostic delay, which can result in substantial morbidity and healthcare costs [1]. Skin manifestations, such as erythroderma, eczematous lesions, or infection, may be present in up to half of these patients at the time of primary immunodeficiency diagnosis [2]. The nature and severity of these clinical manifestations should alert clinicians to the possibility of immunodeficiency [3]. Chronic granulomatous skin lesions can be a presenting feature of major histocompatibility complex (MHC) class I deficiency, a rare autosomal recessive condition caused by mutations in the transporter associated with antigen processing (TAP) [4]. However, the antigenic triggers for the cutaneous granulomas associated with MHC class I deficiency have remained largely elusive. Granulomas associated with rubella virus (RuV) infection have recently been reported in individuals with *TAP1* and *TAP2* deficiency [5, 6]. Here, we report a case of a *TAP1* deficiency diagnosed as a consequence of chronic granulomatous skin ulceration and identify RuV infection as the likely causative agent. To inform diagnosis and management of similar individuals, we perform a scoping literature review assessing the natural history and outcomes of MHC class I deficiency.

## Methods

### Flow Cytometry 

Clinical phenotyping and quantification of MHC class I surface expression were performed on fresh blood using a FACSLyric flow cytometer (BD Biosciences). A healthy age/sex-matched volunteer was included in the analysis for comparison. Descriptive phenotyping of the CD8^+^ T-cell lineage was performed using a custom-built FACSAria II (BD Biosciences). The following antibodies were used in this study: anti-HLA-ABC–FITC (clone G46-2.6, BD Biosciences), anti-CCR7–FITC (clone 150,503, BD Biosciences), anti-CD3–APC/Fire 750 (clone SK7, BioLegend), anti-CD4–PE-Cy5.5 (clone S3.5, Thermo Fisher Scientific), anti-CD8–BV711 (clone RPA-T8, BioLegend), anti-CD14–V500 (clone M5E2, BD Horizon), anti-CD19–V500 (clone HIB19, BD Horizon), anti-CD27–PE-Cy5 (clone 1A4CD27, Beckman Coulter), anti-CD45RA–ECD (clone 2H4, Beckman Coulter), anti-CD57–PE-Cy7 (clone HNK-1, BioLegend), anti-CD95–APC (clone DX2, BioLegend), anti-CD127–PE (clone R34.34, Beckman Coulter), anti-PD-1–BV605 (clone EH12.2H7, BioLegend), and anti-TIGIT–BV421 (clone A15153G, BioLegend). Dead cells were eliminated from the analysis using a LIVE/DEAD Fixable Aqua Dead Cell Stain Kit (Thermo Fisher Scientific). An isotype control antibody (clone MOPC-21, BD Biosciences) was used alongside anti-HLA-ABC–FITC. Data were analyzed using FlowJo version 10.8.1 (FlowJo LLC).

### Whole-exome Sequencing 

Whole-exome sequencing was performed using a Cell3 Target ExomeCG Kit (Nonacus) in conjunction with a NovaSeq 6000 (Illumina). Sequences were aligned to GRCh38. Variant calling was performed using DRAGEN version 3.7 (Illumina) for genes in the NHS R15 Primary Immunodeficiency PanelApp version 2.1.

### Rubella Detection 

RuV capsid (RVC) was detected in histological sections using mouse anti-RVC (clone 9B11, Abcam) and visualized using polyclonal goat anti-mouse IgG–Alexa Fluor 555 (Molecular Probes). Infected cell types were detected in histological sections using rabbit anti-CD206 (clone EPR25215-277, Abcam) or rabbit anti-MPO (clone EPR20257, Abcam) and visualized using polyclonal goat anti-rabbit IgG–Alexa Fluor 488 (Molecular Probes). Nuclei were counterstained with DAPI. Negative and positive control tissue sections were stained in parallel. RT-PCR sequencing of rubella RNA was performed by Micropathology Ltd. (University of Warwick).

### Scoping Literature Review 

We performed a scoping review in order to assess and map the extent of the available evidence, and highlight gaps for future work [7]. PubMed was searched using the terms *(TAP1 OR TAP-1 OR TAP2 OR TAP-2 OR transporter associated antigen processing OR tapasin OR TAPBP OR b2 microglobulin OR b2M) AND (bare lymphocyte syndrome OR MHC I deficiency)* on 23rd August 2023 and updated on 24th April 2025. Abstracts and full texts were screened by reviewer (MJP) against pre-specified eligibility criteria for inclusion (human patients/case reports/literature reviews with a confirmed genetic or functional diagnosis of MHC I deficiency) or exclusion (tumor cell lines/animal-only models/unreported clinical information). This approach was complemented by a bibliographic review of included articles and search results from the human genome mutation database (https://www.hgmd.cf.ac.uk/) and online mendelian inheritance in man (OMIM, https://omim.org/) for *TAP1*, *TAP2*, tapasin (*TAPBP)*, and β2-microglobulin (*B2M*) deficiency associated with the bare lymphocyte syndrome phenotype. Eligibility queries were resolved in consultation within team. Pre-defined clinical, genetic, and laboratory data were extracted to Excel for narrative synthesis (Supplementary S2). We aimed to characterise the state of knowledge on natural history of MHC class I deficiency, with the pre-specified objectives: (i) describe the demographics, clinical presentation, age at presentation (taken as the first infectious or cutaneous presentation), typical diagnostic delay, ethnicity, and history of consanguinity; (ii) summarize the genetic diagnosis and immunological features (including residual MHC class I expression and CD8^+^ T-cell count); (iii) characterize the age of onset for bronchiectasis and cutaneous ulceration and the potential relationship to overall survival at last follow-up; and (iv) summarize the reported approach to clinical care (including infectious complications, treatment of cutaneous lesions, and outcomes of bone marrow transplantation). We made the following assumptions with regards to missing data to better estimate diagnostic delay or survival: where age at diagnosis was stated as “childhood”, this was handled as 10 years. Where the age of one individual described as an elder sibling [8], we used a conservative estimate for age at last follow-up in survival analysis (one year older than the age of the younger sibling). The absence of a documented clinical finding (e.g., bronchiectasis) was taken as a negative. Narrative synthesis was supported by exploratory Kaplan–Meier survival curve plotting using the *survminer* package [9] in R version 4.0.5 and RStudio version 1.3.959.

## Results

### Case Report

**Fig. 1:**
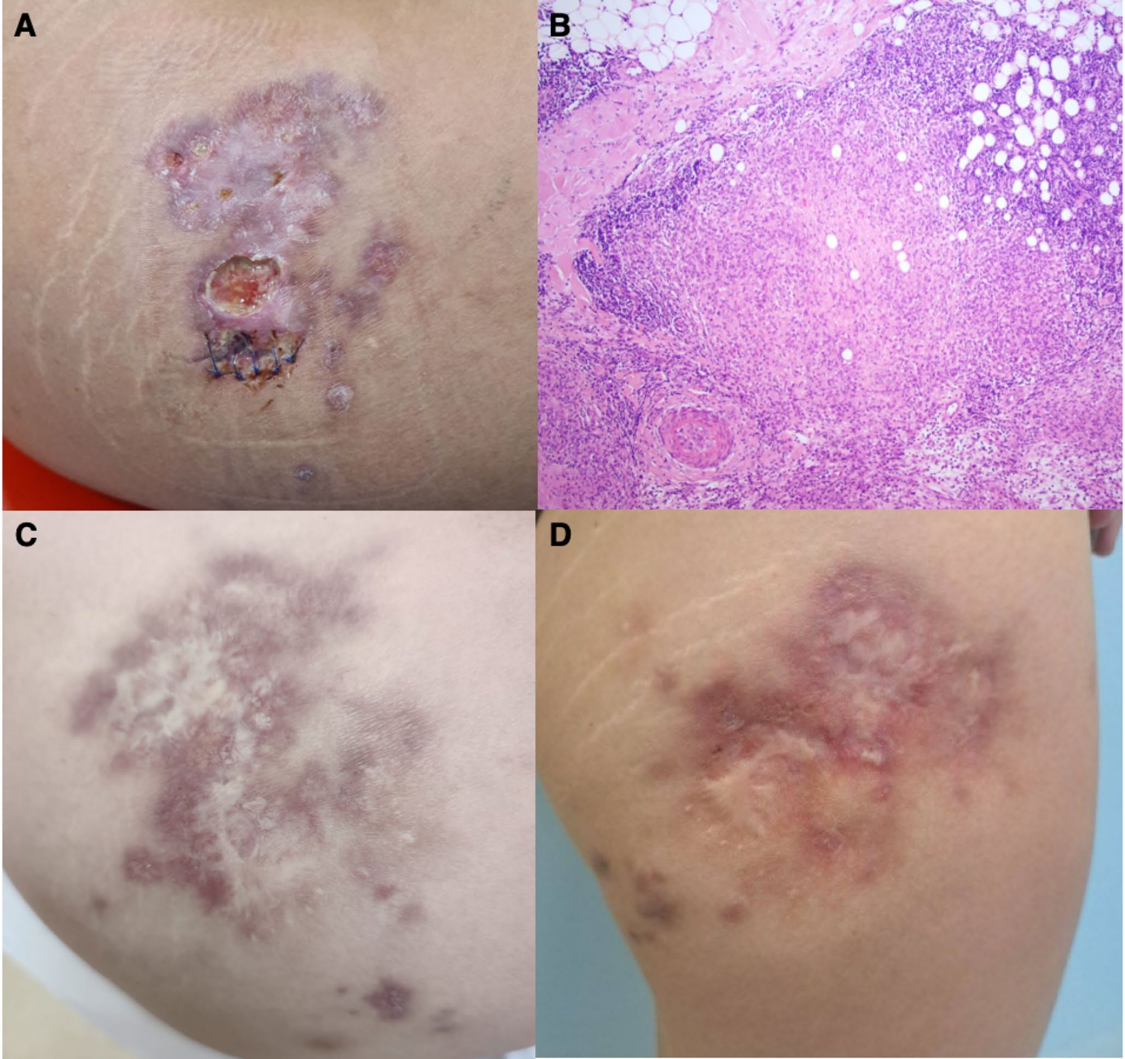
Clinical and histological features of *TAP1* deficiency. (**A**) Cutaneous ulcer on right thigh 5 years after onset. (**B**) Histology of the granulomatous plaque showing deep necrotizing epithelioid granulomatous inflammation within the dermis and subcutaneous tissue. The granulomas were surrounded by a dense chronic inflammatory cell infiltrate composed of lymphocytes. Histochemical staining for fungi, mycobacteria and microorganisms was negative. (**C**, **D**) Modest improvement after 3 months of topical tacrolimus (0.1%) was sustained at 6 months (**C**) and 10 months after discontinuation of therapy (**D**)

A 25-year-old Spanish female presented with a cutaneous nodule over her right thigh, which ulcerated and enlarged slowly (Fig. 1A). Over the following 7 years, she underwent multiple skin biopsies for necrotizing granulomatous inflammation, but investigations for fungal, mycobacterial, and leishmanial pathogens failed to identify a causative organism (Fig. 1B). Serum angiotensin-converting enzyme and autoantibody (ANA, ANCA, and ENA) concentrations were within normal limits. She tested negative for TB by interferon (IFN)-γ release assay, and serology for toxoplasma and HIV were negative. After specialist dermatology, tropical medicine, and infectious disease review across multiple centres, she was referred for evaluation of possible underlying immunodeficiency at 32 years of age.

She required approximately monthly courses of antibiotics from 7 to 14 years of age and required two admissions to hospital for respiratory tract infections and possible bronchiectasis on chest imaging during this time. Investigations for cystic fibrosis and primary ciliary dyskinesia were unremarkable, and she received allergen immunotherapy in childhood with olive tree pollen and *Alternaria*. Her infection burden improved during adulthood, and at the time of immunological assessment, she described ongoing sinus congestion and a postnasal drip, but had not required antibiotics over the 12 months to Immunology assessment. Computed tomography imaging showed mucosal thickening in a single left posterior ethmoid air cell but otherwise clear sinuses and no evidence of bronchiectasis. Her father died of motor neurone disease, and her mother suffered venous thromboses with a diagnosis of Factor V Leiden. There was no history of consanguinity, although both parents originated from the same village, which had a population of approximately 3,000.

**Fig. 2:**
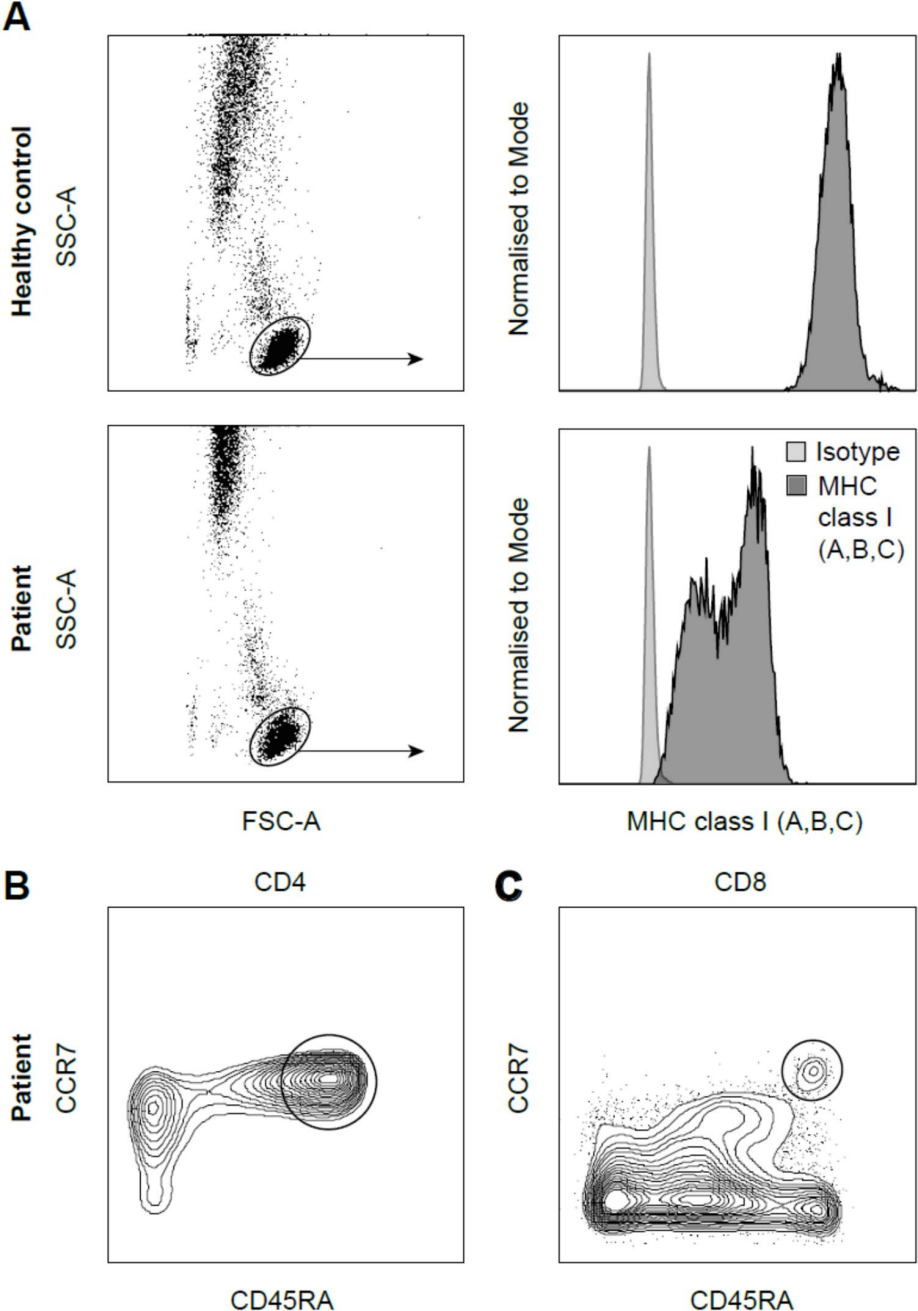
Immunological features of *TAP1* deficiency. (**A**) Representative flow cytometry plots showing the gating strategy and histograms for surface MHC class I expression (dark gray) in a healthy donor (top) and the patient (bottom) relative to an isotype control (light gray). (**B**, **C**) Representative flow cytometry plots showing the frequencies of naive (CCR7^+^CD45RA^+^) CD4^+^ T-cells (**B**) and CD8^+^ T-cells (**C**)

Immunological evaluations revealed a reduced CD8^+^ T-cell count (160 × 10^6^ cells/L) for age (normal range = 200–1,100 × 10^6^ cells/L) with a skewed CD4:CD8 ratio of 8.5 (normal range = 0.70–3.10), but otherwise normal lymphocyte subset counts and serum concentrations of IgG, IgA, and IgM. A markedly reduced frequency of naive CD8^+^ T-cells (18% of total CD8^+^, 29 × 10^6^ cells/L) was noted relative to naive CD4^+^ T-cells (49% of total CD4^+^, 670 × 10^6^ cells/L), equating to a naive CD4:CD8 ratio of 30.1 (Fig. 2). Whole-exome sequencing identified a homozygous variant in the *TAP1* gene (NM_000593.6:c.1564C > T; NP_000584.3:p.(Gln522Ter)), expected to cause nonsense-mediated decay of the mRNA transcript of the *TAP1* gene, resulting in reduced expression of the TAP-1 protein [10]. Flow cytometric analysis confirmed a tenfold reduction in surface MHC class I expression on peripheral blood lymphocytes relative to an age-matched healthy control (Fig. 2), consistent with previous characterization of this mutation [8]. Extended immunophenotyping suggested that CD4^+^ T-cell subpopulations (Th1, Th2, Th17, and Treg) were largely preserved (data not shown), again consistent with a previous study [4]. Descriptive multiparameter assessment of the CD8^+^ T-cell lineage is presented in Supplementary Figure S3-1. Here, a small population of naive cells (CCR7^+^ CD27^+^ CD45RA^+^ CD95^−^) was accompanied by a much larger population of memory cells (CD95^+^), many of which exhibited an early differentiation phenotype (CD27^+^ CD57^−^ CD127^+^), consistent with limited antigen exposure. A subpopulation of precursor exhausted memory cells was also notable, characterized by the expression of CCR7, PD-1, and TIGIT.

**Fig. 3:**
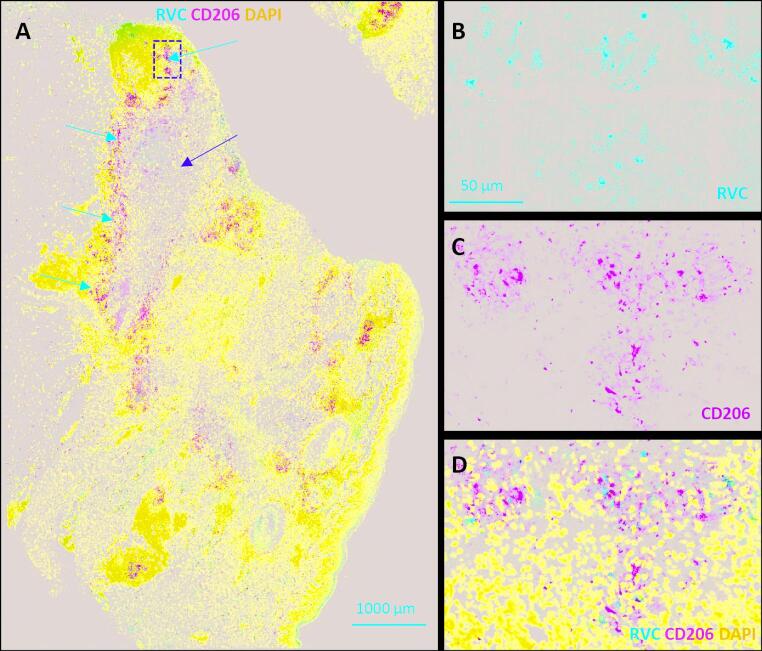
Double immunofluorescent staining of FFPE skin punch biopsy sample. The formalin-fsixed paraffin-embedded (FFPE) section was stained with mouse monoclonal antibody against rubella virus capsid (RVC, red) and rabbit polyclonal antibody against CD206, an M2 macrophage marker (green). Nuclei were counterstained with DAPI (blue). (**A**) Overview of biopsy showing large necrotic area (yellow arrow) surrounded by M2 macrophages (red arrows). Punctuate RVC staining is primarily localized in CD206^+^ macrophages surrounding the necrotic zone. A small number of MPO.^+^ neutrophils were also detected (not shown). (**B**-**D**) Higher magnification of the region indicated by the yellow dashed box in (A): (**B**) RVC channel; (**C**) CD206 channel; (**D**) merged image including DAPI. Scale bars: 1000 μm (**A**) and 50 μm (**B**)

*Staphylococcus aureus* was cultured on several occasions from the ulcer, but treatment with antibiotics was associated with limited improvement, suggesting an alternative driver of tissue inflammation. The presence of a violaceous skin rash associated with a chronic necrotizing skin lesion at a common site of childhood vaccination in the context of immunodeficiency led us to consider the possibility of RuV infection [11, 12]. Immunofluorescence staining of lesional tissue revealed the presence of rubella virus capsid protein, which colocalized with M2 macrophages surrounding areas of necrosis (Fig. 3). Rubella RNA was confirmed within tissue on RT-PCR, and RuV-specific IgG was also detected in serum. Rubella RNA was not detectable on a throat swab. Topical steroid therapy was not beneficial after 6 months. Twice daily topical application of 0.1% tacrolimus ointment was trialed following reports of benefit in chronic granulomatous dermatoses [13]. Modest improvement associated with re-epithelialisation was observed after 3 months, although new violaceous satellite lesions were also apparent, suggesting ongoing infection or inflammation at 6 months after discontinuation of therapy (Fig. 1C). At time of submission, clinical follow-up extended to 10 months post-tacrolimus with sustained benefit (Fig. 1D).

### Scoping Literature Review

To better inform the clinical management of such individuals, we undertook a scoping literature review of NCBI Medline with a focus on clinical reports describing outcomes for genetically confirmed cases of MHC class I deficiency. We followed the PRISMA extension for scoping reviews checklist [14] (Supplementary Materials S1). PubMed search terms returned 442 results on 24th April 2025, with an additional 55 articles identified from human genome mutation database and online mendelian inheritance in man. Abstracts were screened against prespecified eligibility criteria, identifying 48 articles for full text review. This approach was supplemented by bibliographic review of included articles and a search of human genome mutation and OMIM databases to identify reports of *TAP1*, *TAP2*, *tapasin* (*TAPBP*), and *B2M* mutations associated with MHC class I deficiency. We identified 45 unique individuals from 36 reports a combined follow-up duration of 1184 patient years (see online Supplementary Table S2). This included deficiency of *TAP1* (*n =* 20, including the present case) [8, 15–27], *TAP2* (*n =* 19) [4, 24, 28, 28–37], combined *TAP1*/*TAP2* (*n =* 2) [38, 39], *TAPBP* (*n =* 2) [40, 41], *B2M* (*n =* 2) [42].

### Individuals With MHC Class I Deficiency are Typically Diagnosed a Decade After Symptomatic Presentation

Given the diagnostic odyssey spanning almost two decades experienced by our patient, we first sought to assess the delay for other individuals with MHC class I deficiency. Median age at diagnosis of MHC I deficiency was approximately 21 years. Age at initial clinical presentation was available for 30/45 individuals and ranged from 6 months to 44 years (median age = 9 years), with a typical delay of 11 years (range = 1–33 years) from the onset of clinical symptoms (data available for 30/45 cases, Table 1). Four adults from 2 unrelated families with genetically-confirmed TAP2 deficiency remained asymptomatic at the time of diagnosis (median age = 32 years, range = 28–40 years). Investigation in these cases was prompted by an equivalent diagnosis in a symptomatic family member [4, 36].

**Table 1:** Summary of characteristics for reported cases of MHC class I deficiency

Characteristic	Median, years	Range, years	Data completeness
Age at last follow-up	27	3–61	45/45 (100%)
Age at diagnosis	21	3–61	44/45 (98%)
Age at first symptoms	9	0.5–44	30/45 (67%) (*n =* 4 asymptomatic)
Diagnostic delay	11	1–33	30/41(73%)
Age at onset cutaneous ulceration	11	1–33	23/45 (51%)
Age at onset bronchiectasis	15	6–39	24/45 (53%)
	**Present**	**Absent**	**Data completeness**
History of known consanguinity	20/45 (44%)	25/45 (%)	45/45* (100%)
Family history recurrent or atypical infections	27/45 (60%)	18/45 (40%)	45/45* (100%)
CD8^+^ T-cell lymphopenia	15/32 (47%)	17/32 (53%)	32/45 (71%)
Cutaneous ulceration	26/45 (58%)	19/45 (42%)	45/45* (100%)
Bronchiectasis	25/45 (56%)	20/45 (44%)	45/45* (100%)
All cause mortality	5/45 (11%)	40/45 (89%)	45/45 (100%)

^*^The absence of a reported finding was taken as negative for these fields

To explore potential contributors to this diagnostic delay across MHC I deficiency, we next examined the frequency of commonly used “red flags” for immunodeficiency, including personal and familial history of severe or atypical infections, consanguinity, or the presence of CD8^+^ T-cell lymphopenia in the context of MHC class I deficiency. Details of clinical symptoms prompting medical attention are summarized in Table 2. Recurrent sinopulmonary infections or cutaneous ulceration accounted for the initial presentation in a majority of cases (81%). A family history of severe or recurrent infections was noted in 27/45 cases (60%), with high rates of known consanguinity in 20/45 cases (45%). Two individuals were diagnosed following failure of serological typing for MHC class I, requested during workup for lung or renal transplantation prompted by recurrent pulmonary infections or idiopathic chronic glomerulonephritis [31, 32, 40].

**Table 2:** Summary of initial clinical features of MHC class I deficiency

Initial presenting feature	Frequency (%)
Recurrent sinopulmonary infections	20 (45%)
Cutaneous ulceration	12 (27%)
Cutaneous and sinopulmonary infections	4 (9%)
Meningitis	2 (4%)
Recurrent otitis media and neutropenia	1 (2%)
Ocular toxoplasmosis	1 (2%)
Chronic glomerulonephritis prompting renal transplant evaluation	1 (2%)
Asymptomatic	4 (9%)

Lymphocyte subset data were reported in 32/45 cases, and reduced CD8^+^ T-cell counts for age were detected in 15/32 cases, equating to a sensitivity of approximately 47%. Several groups noted that CD4^+^ and CD8^+^ T-cell lymphopenia developed progressively [20, 26]. Expansion of the γδ CD8^+^ T-cell compartment was described in the context of *TAP2* [36] and *B2M* deficiency [42] but was not observed universally [16, 29]. Darazam et al*.* recently performed deep immunophenotyping of a family with *TAP2* deficiency [4]. In line with our report, they identified reduced naive CD8^+^ T-cell frequencies in two individuals with genetically confirmed *TAP2* deficiency and normal CD8^+^ T-cell counts for age [4]. Reduced naive CD8^+^ T-cell counts were also noted in two adults within this study, including an asymptomatic adult [4]. Consistent with our results, the naïve CD4/CD8 ratio was strongly increased relative to controls [4]. This suggests diagnostic delay is common for individuals with MHC Class I deficiency, and likely reflects a combination of broad clinical expressivity, disease rarity, and limited sensitivity of red flags for primary immunodeficiency such as consanguinity. Notably, a low CD8 + T-cell lymphocyte count offered low sensitivity for the detection of MHC Class I deficiency. Together, this highlights the use of naïve T-cell enumeration, naïve and total CD4/8 ratio, MHC class I evaluation, and access to clinical genetic sequencing in diagnosis of this rare condition.

### Overall Survival, Bronchiectasis, and Residual MHC Class I Expression

**Fig. 4:**
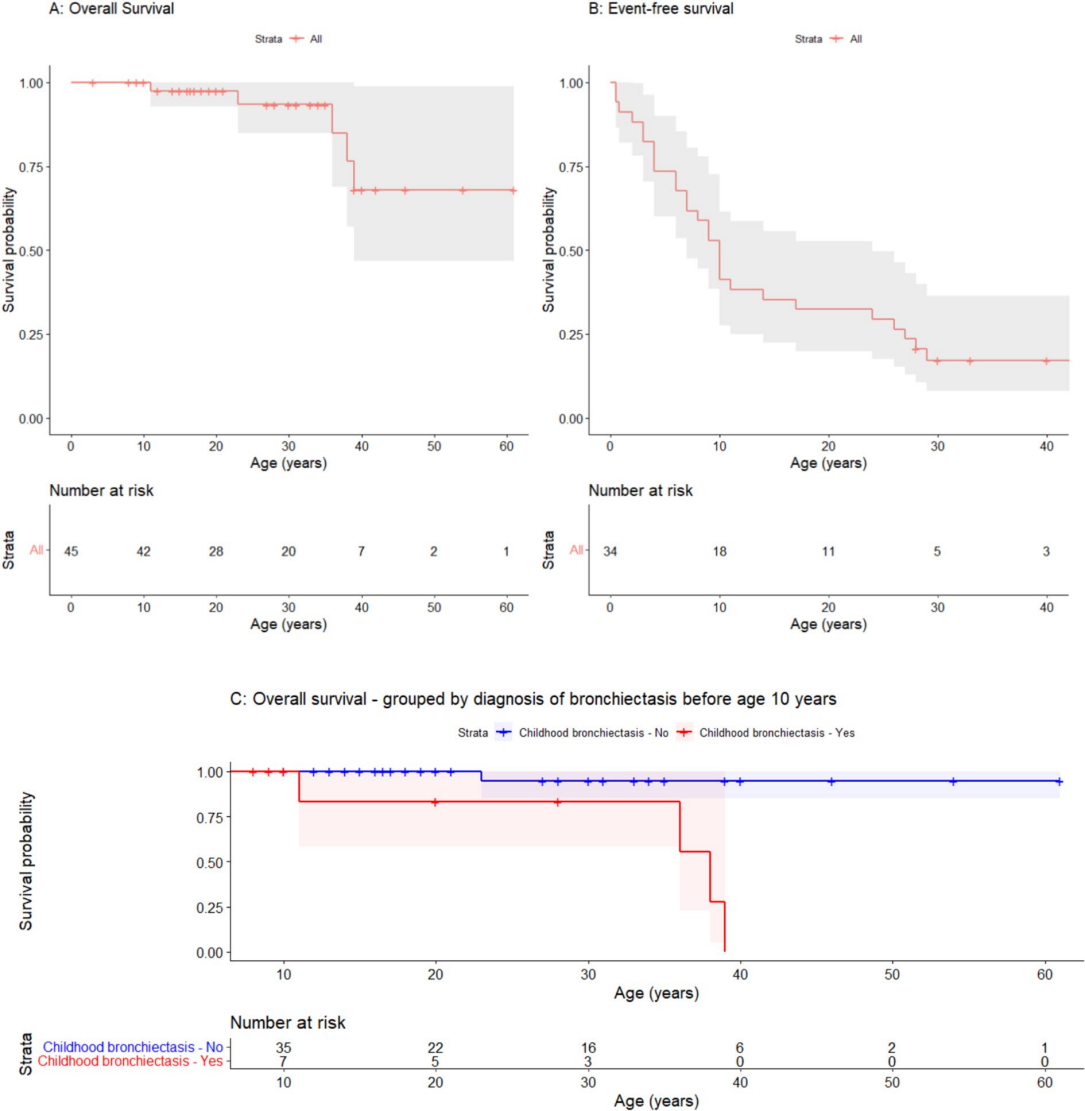
Survival estimates for individuals with MHC class I deficiency. (**A**) Kaplan–Meier plot showing the probability of survival for individuals with MHC class I deficiency. (**B**) Kaplan–Meier plot showing the probability of symptom-free (event-free) survival for individuals with MHC class I deficiency. (**C**) Kaplan–Meier plot showing the probability of survival for individuals with MHC class I deficiency, stratified by the diagnosis of bronchiectasis before the age of 10 years. To mitigate potential survivorship bias, only outcomes following 10 years of age are considered in this sub-analysis. Shaded areas indicate 95% confidence intervals

Age at last follow-up was available in 45/45 cases (media*n =* 27 years, range = 3–61 years). Kaplan–Meier plots for overall survival are shown in Fig. 4A, indicating similar trajectories for *TAP1* and *TAP2* deficiency (Supplementary Figure S3-2). Five individuals were reported to have died (median age = 36 years, range = 11–39 years). Four deaths occurred as a result of recurrent infections and respiratory failure [21, 24, 31, 32], including one after allogeneic stem cell transplantation [26]. One individual with chronic skin ulceration since the age of 9 years experienced malignant transformation to Marjolin’s ulcer and died as a consequences of metastatic disease, despite limb amputation [29].

Individuals with MHC class I deficiency manifested their first reported symptoms across a broad range of ages (Fig. 4B). Bronchiectasis was reported in 25/45 cases (56%) and occurred by the age of 10 years in 9/25 cases (36%) (Supplementary Figure S3-3). The development of bronchiectasis during childhood appeared closely associated with the risk of subsequently mortality (Fig. 4C). No relationships were detected between overall survival and residual MHC class I expression, development of bronchiectasis, or the presence of CD8^+^ T-cell lymphopenia (data not shown). This finding is consistent with phenotypic data showing that comparable reductions in MHC class I expression levels occur in asymptomatic and symptomatic individuals and in 5–15% of healthy individuals when using a standardized flow cytometric approach [4].

### Nature and Treatment of Chronic Cutaneous Ulceration

Chronic cutaneous ulceration was reported in 26/45 cases (58%), with granulomatous inflammation described in 23/26 cases (88%). Age at onset was available in 23 cases, with an approximate median age of 12 years (range = 2–43 years). No association was detected between overall survival and the development of cutaneous ulceration (Supplementary Figure S3-4). Cutaneous lesions were misdiagnosed variously as sarcoidosis [4], granulomatosis with polyangiitis (formerly Wegner’s granulomatosis) [16], and seronegative vasculitis [24]. Despite extensive investigations, a microbiological cause for cutaneous ulceration was suggested in only five individuals, including three with positive deep-wound cultures for *Staphylococcus aureus* [26, 29, 30], one where human herpesviruses and Ebstein-Barr virus were detected via PCR [38], and one with suspected toxoplasmosis based on positive IgM serology [15]. In this latter case, therapy for toxoplasmosis alongside IgG replacement was associated with regression of the skin ulcers and resolution of an acute pulmonary infection [15]. Antimicrobial therapy for mycobacterial [22, 24, 42] and other bacterial infections [15, 25, 26] was commonly used but generally ineffective. One instance of remission of a chronic lower limb ulcer was described in a 47-year-old male with *TAP1* deficiency, albeit 4 months after completion of a 9-month course of empirical antituberculosis therapy [36].

Eight individuals developed erosive midline granulomatous lesions [4, 22, 24, 26, 42], with at least five individuals receiving systemic immunosuppression (cyclophosphamide and/or high-dose corticosteroids). Systemic immunosuppression did not generally appear associated with improvement of cutaneous features, but was accompanied by clinical deterioration, including the development of bronchiectasis [16, 22, 24]. In contrast, Bhattarai et al. recently reported favourable response of lower limb necrotising granulomas to prednisolone and cyclosporin [43], although the lesions remained. Law-Ping et al*.* trialled prolonged clarithromycin and chloroquine therapy, but no improvement was observed clinically [25]. A single case report described ulcer healing following allogeneic haemopoietic stem cell transplantation (HSCT), with post-transplant survival follow-up extending to 15 years at the time of publication [38]. The only other individual reported to have undergone allogenic HSCT (aged 11 years) developed severe graft-versus-host disease and pneumonitis associated with CMV and parainfluenza II viral infections, leading to death from multiple organ failure 69 days after transplantation [26]. Therapy with IFN-α or IFN-γ was described in three individuals [5, 24, 26], but was associated with lesion progression. Wang et al. recently described a similar case of chronic granulomatous inflammation associated with rubella viral infection and TAP1 deficiency, here intralesional IFN-α2b and topical TLR-7 agonist therapy (imiquimod) were tried, but surgical resection was felt to offer a more favourable treatment [5].

### Low Incidence of Systemic Viral Infections

Previous reviewers have suggested episodes of measles and chickenpox were typically unrecorded or uneventful clinically [24]. However, two recent reports of MHC class I deficiency have documented systemic viral infections including hepatitis B viremia associated with transaminitis [23], and disseminated herpes viral infections requiring antiviral therapy [43]. It is possible that increasing accessibility of molecular diagnostic methods may reveal a greater burden of systemic viral infections in MHC I deficient individuals.

### Potential Unreported Cutaneous Rubella Virus Infections in Cases of MHC Class I Deficiency

RuV infection associated with MHC class I deficiency was not identified during our initial literature review. Three cases of RuV-associated cutaneous granulomas have recently been reported to date in the setting of *TAP1* and *TAP2* deficiency [5, 6]. We therefore screened published images and case descriptions for the presence of violaceous plaques and ulceration, which are common features of chronic cutaneous rubella infection [12]. We identified one case with chronic violaceous skin ulceration affecting the buttocks of a child with *TAP1* deficiency [25] and at least two additional cases describing chronic violaceous skin ulceration affecting the mouth and nose [15, 42]. These represent common sites for childhood RuV vaccination or replication, respectively.

## Discussion

Here, we report a case of MHC class I deficiency caused by a homozygous mutation in *TAP1*, diagnosed 7 years after the onset of a cutaneous ulcerating granulomatous skin lesion at a common site of childhood vaccination. RVC was detected in the lesion using direct immunofluorescence and RT-PCR, confirming a hypothesis first proposed by Tsilifis et al*.* [26]. A trial of the topical calcineurin inhibitor tacrolimus has been associated with healing but not complete resolution of the lesion. These observations parallel reports of chronic RuV infection associated with cutaneous granulomatous ulceration in individuals with *TAP1* and *TAP2* deficiency [5, 6].

Little is known about the natural history of MHC class I deficiency. We therefore set out to provide a overview of this rare condition via a systematic scoping review. We found that chronic necrotizing granulomatous skin lesions and childhood-onset bronchiectasis were common but not universal clinical features of *TAP1*, *TAP2*, *TAPBP*, and *B2M* deficiency. At least four individuals reached adulthood without clinical complications, suggesting incomplete penetrance. In symptomatic cases, diagnostic delay frequently exceeded a decade. Misdiagnosis of granulomatous lesions during this time was associated with use of systemic immunosuppression and infection-related morbidity. Using a systematic approach, we identified 45 individuals with genetically confirmed MHC class I deficiency reported over four decades (1985–2025), representing the most comprehensive review assembled to date.

Genotype–phenotype correlations have been postulated by Bhattterai et al. in the setting of TAP1 deficiency [43]. Some features emerge within our present report, including the presence of hypoalbuminaemia and panhypogammaglobulinaemia with β2 microglobulin deficiency [42] that distinguish it from other causes of MHC I deficiency. However the current sample size nonetheless introduces caveats to interpretation. For instance, whilst all 4 asymptomatic individuals carried TAP2 mutations, the variable penetrance of MHC I deficiency suggests that undiagnosed asymptomatic TAP1 deficiency also exist. We found that a diagnosis of bronchiectasis during childhood was associated with a greater risk of mortality, which is consistent with the high rate of mortality attributed to recurrent pulmonary infection (accounting for four of the five reported deaths). Here, the potential for publication bias and variable follow-up mean this finding should be regarded with caution. It was also not possible to address potential confounders of clinical severity. For instance, consanguinity was common, potentially contributing to phenotypic complexity. Finally, residual MHC I expression was inconsistently reported (including variation in use of cell-lines, unsorted and sorted peripheral blood lymphocytes), which may have limited appreciation of this as a prognostic indicator. Our findings nonetheless have several immediate implications for clinicians. In particular, we examined the sensitivity of CD8^+^ T-cell lymphopenia as a diagnostic indicator of MHC class I deficiency, which would be predicted to impact thymic selection. Remarkably, almost half of the individuals tested were found to have normal CD8^+^ T-cell counts for age, indicating that a normal lymphocyte count should not deter further investigation. A low threshold for naive T-cell enumeration, review of naive and total CD4:CD8 ratios, genetic sequencing, and MHC class I expression studies would therefore be advisable to ensure timely diagnosis and direct appropriate therapy [4].

Our findings further suggest that the phenomenon of chronic RuV infection is likely underreported in cases of MHC class I deficiency. The graphical review of case reports undertaken here identified at least three such individuals with violaceous skin lesions that were morphologically similar to known manifestations of RuV-associated cutaneous disease [15, 25, 42]. Indeed, during the course of the literature review, a parallel case of RuV-associated cutaneous granulomas in an individual with *TAP1* deficiency was reported by Wang et al. [5]. We therefore suggest that MHC class I deficiency is part of an emerging spectrum of Mendelian disorders characterized by susceptibility to chronic infection with RuV [6].

Vaccine-strain RuV has been found to persist for decades before emerging in granulomas in the context of immunodeficiency [11]. The extent to which RuV remains infectious in this setting remains uncertain, with the potential risk of congenital infection a particular consideration in the present case. Although outcome surveillance appears reassuring with respect to the risk of symptomatic congenital infection following inadvertent immunization of unvaccinated women [44], genomic sequencing of RuV obtained from granulomas affecting individuals with inborn errors of immunity reveals ongoing viral evolution [45]. Broad-spectrum antiviral therapies and immunoglobulin replacement, which delivers high levels of neutralizing antibodies, have not proven beneficial in immunodeficient individuals with chronic RuV infection [11, 45]. Accordingly, our patient is undergoing further clinical evaluation and is currently under consideration for possible HSCT as the only reported option for clearance of chronic rubella virus-associated granulomas in the setting of primary immunodeficiency [11, 46]. Given the history of rubella vaccination, sequencing has not been performed to date.

In summary, we suggest that RuV testing should be considered in individuals with skin lesions and a diagnosis of MHC class I deficiency. Moreover, we propose that the finding of chronic granulomatous ulceration associated with RuV infection in seemingly immunocompetent adults, as reported recently [47], should prompt further evaluation for MHC class I deficiency. The natural history and optimal management strategy for individuals with MHC class I deficiency remains unclear at the present time. We therefore advocate an international registry survey to better understand the contribution of chronic viral infections to cutaneous lesions in individuals with MHC class I deficiency. Ultimately, novel therapeutic approaches, such as combined antiviral and immunomodulatory protocols or therapeutic vaccination [48], may be required to optimize the management of immunodeficient individuals persistently infected with RuV.

## Supplementary Information

Below is the link to the electronic supplementary material.

Supplementary file1 (XLSX 28.6 KB)

Supplementary file2 (PDF 229 KB)

Supplementary file3 (DOCX 7.56 MB)

## Data Availability

No datasets were generated or analysed during the current study.
