# Supplementary material for: TAP-I Deficiency Presenting With Chronic Granulomatous Rubella Virus-Driven Cutaneous Ulceration: A Case Report and Scoping Literature Review
Source: J Clin Immunol. 2025 Nov 27;46(1):1. doi: 10.1007/s10875-025-01919-6 (PMC12774932; doi:10.1007/s10875-025-01919-6)

**Supplementary Figure 3-1: Deep immunophenotyping of CD8^+^ T-cells in a patient with *TAP1* deficiency.** Uniform Manifold Approximation and Projection (UMAP) plots showing the distribution of immunophenotypic markers among circulating CD8^+^ T-cells from a patient with *TAP1* deficiency.

**
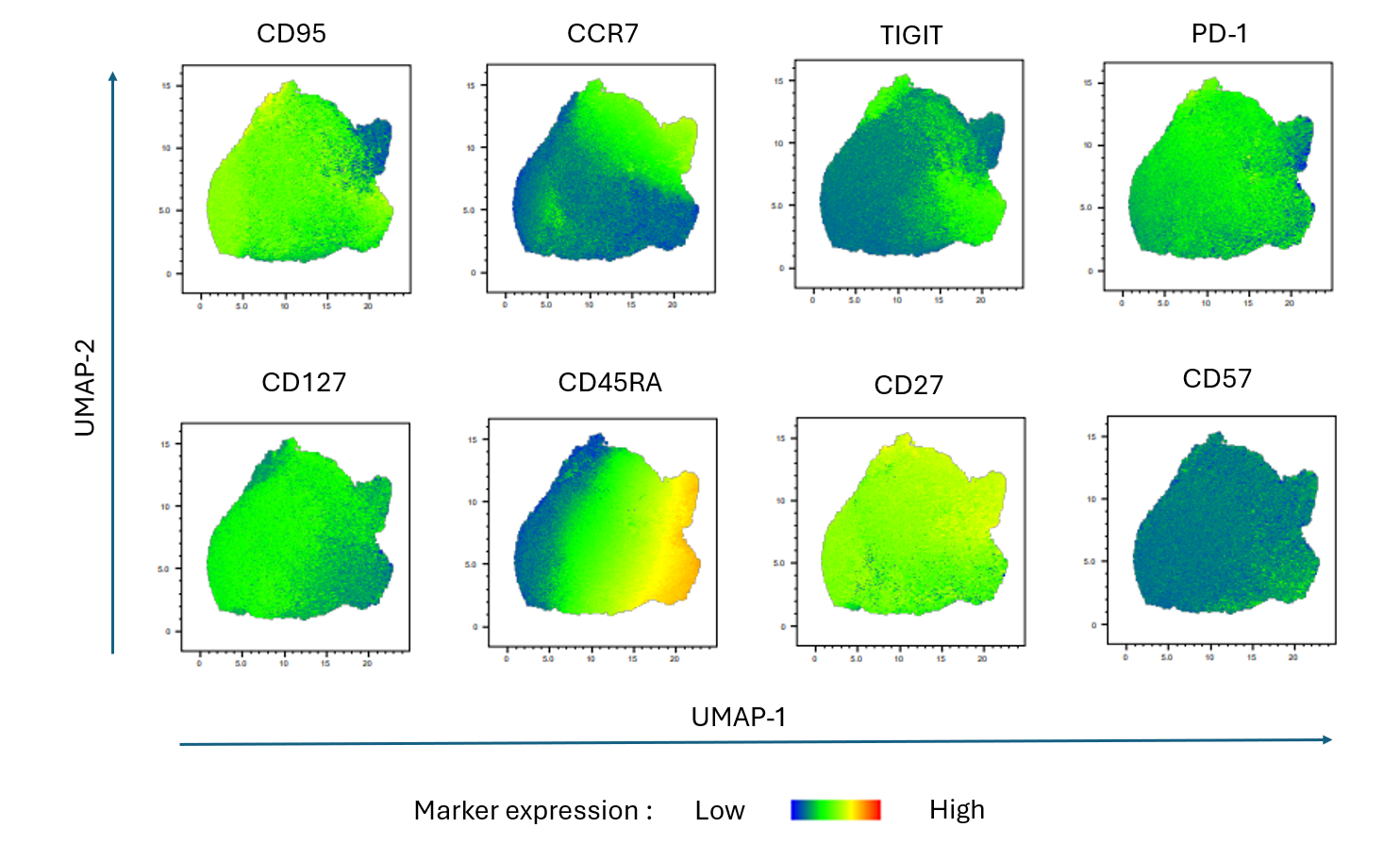
**

**Supplementary Figure 3-2: Survival estimates for individuals with distinct forms of MHC class I deficiency.** Kaplan–Meier plot showing the probability of survival for individuals with MHC class I deficiency stratified by gene defect: *TAP1* (green); *TAP2* (blue); combined *TAP1/TAP2*, *TAPBP*, and *B2M* (red). Shaded areas indicate 95% confidence intervals.


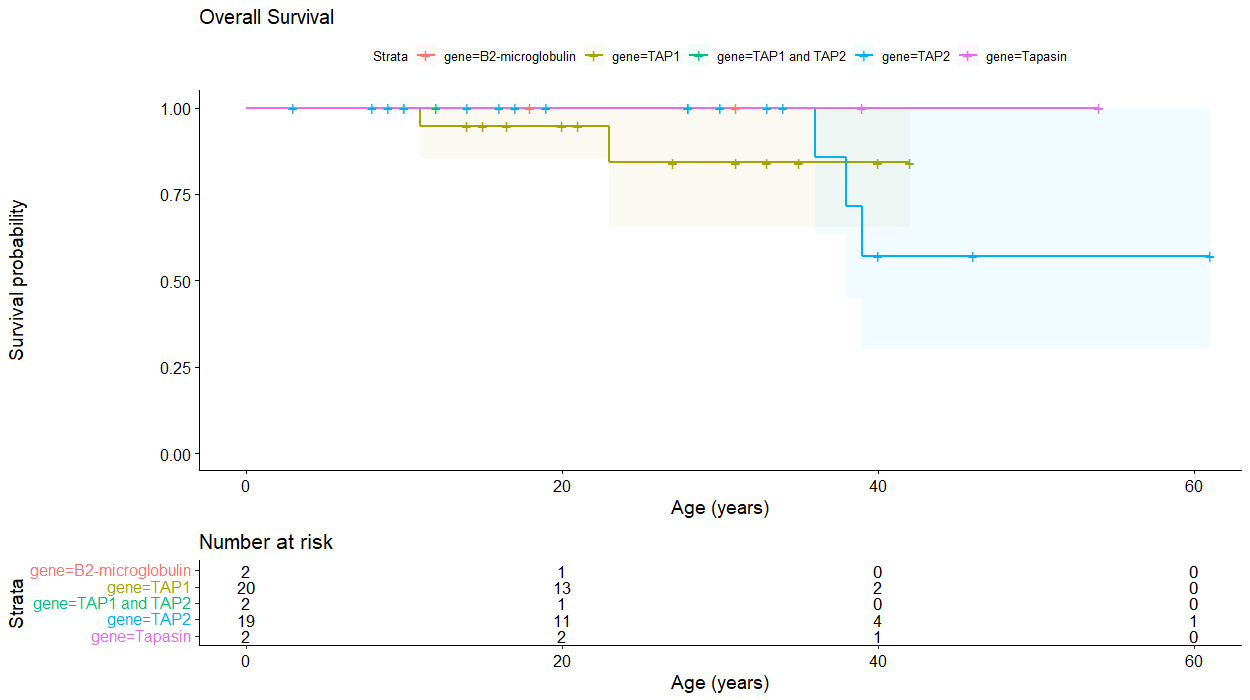


**Supplementary Figure 3-3: Bronchiectasis-free survival estimates for individuals with MHC class I deficiency.** Kaplan–Meier plot showing the probability of bronchiectasis-free survival for individuals with MHC class I deficiency.


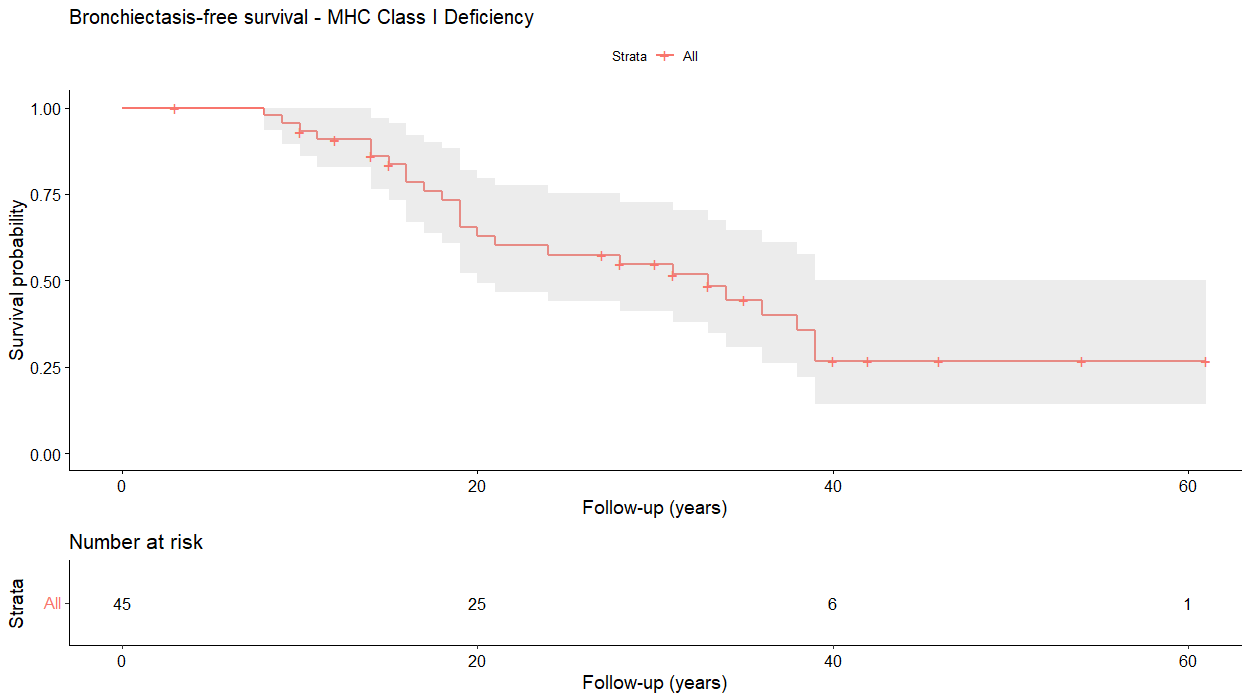


**Supplementary Figure 3-4: Survival estimates for individuals with or without cutaneous lesions associated with MHC class I deficiency.** Kaplan–Meier plot showing the probability of survival for individuals with MHC class I deficiency stratified by the presence of cutaneous lesions. Shaded areas indicate 95% confidence intervals.


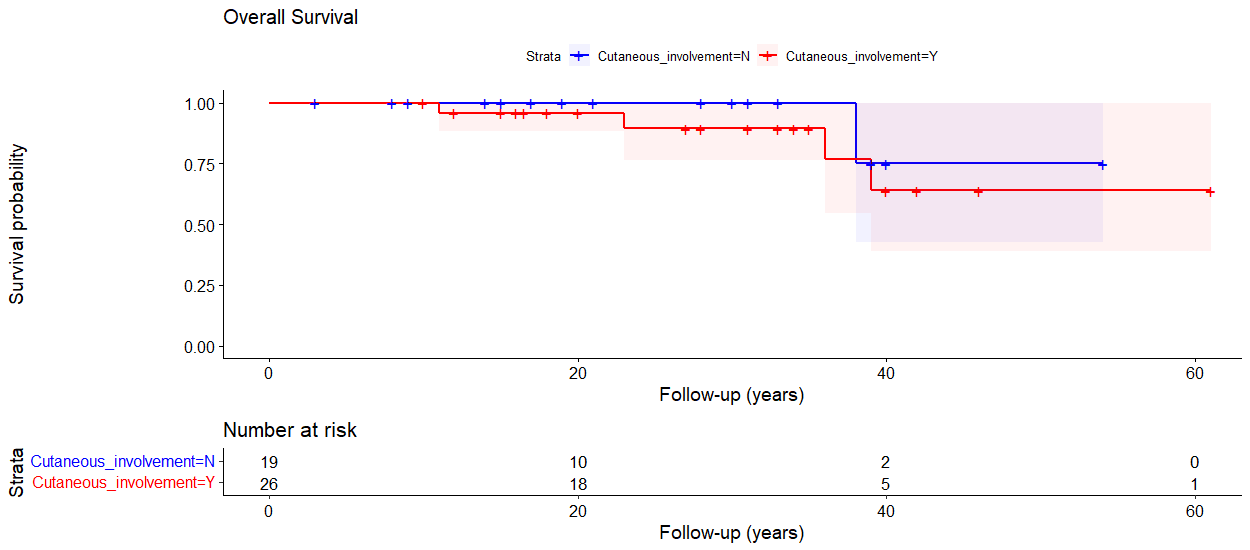

Supplement: Supplementary file 3 — Supplementary file3 (DOCX 7.56 MB) [file 10875_2025_1919_MOESM3_ESM.docx]
